# Sulphate of Magnesia

**Published:** 1907-02

**Authors:** 


### EDITORIAL NOTES AND COMMENTS

The Business Office of the JOURNAL-RECORD is 1007-1008 Century Building.

The Editorial Office is 318-319-320 The Prudential.

Address all Business Commucications to Dr. M. B. Hutchins, Manager.

Make remittances payable to THE ATLANTA JOURNAL-RECORD OF MEDICINE.

On matters pertaining to the Editorial and Original communications, address Dr, Bernard Wolff, Atlanta.

Reprints of original articles will be furnished at cost price. Requests for the same should always be made on THE MANUSCRIPT.

We will present, postpaid, on reqnest, to each contributor of an original article, twenty (20) marked copies of THE JOURNAL-RECORD containing such article.

### SULPHATE OF MAGNESIA.

The late Dr. Virgil O. Harden used to remark that if he were to be deprived of every remedy but one, he would select Epsom salts as that one. When one contemplates the variety of affections encountered in the general practice of medicine in the treatment of which he recommends a dose of salts, he will willingly accord to this ancient and prosaic remedy a well nigh indispensable position in practical therapeutics. Clinicians have come more and more to regard the intestinal canal from some defect in the elaborate chemistry by which the qualities of the ingesta are adapted to the purposes of the body, as the fountain source of much evil. And as in the chemical laboratory when an experiment has failed, the beakers and retorts are cleansed by a copious drenching of top water, the alimentary tube is scoured out by a perfect Niagara, loosened by a heavy charge of salts, and the fermenting debris of a digestice failure is swept by easy stages into the sewer.

Apart from the purgative effect of magnesium sulphate o rof sodium sulphate, some interesting facts concerning its further action are brought out in an article by Maberly in the *London Lancet*, the salient points having been abstracted by the *Medical Record*, as follows:

"Some suggestive remarks are made by J. Maberly in the *Lancet*

of November 10th, regarding the true antiseptic action of sodium sulphate in intestinal toxæmia. Happening to employ the remedy in cases of dysentery, with a view to free but gentle purgation, he noticed that in the dosage ordinarily used for such purgation the latter was not necessarily produced. In fact, in the most successful cases the evacuations became less frequent and assumed their normal color and consistency. These results led him to believe that the action of the remedy under the conditions named was referable to qualities other than purgative. He, therefore, began to use the drug in cases of infantile diarrhœa, and found that if the dose was reduced below the aperient effect the evacuations became less frequent, and often assumed a normal character without other medication. Also it was observed that vomiting quickly ceased. Milk diet was, of course, avoided as much as possible. Since obtaining such results he has relied more and more on sodium sulphate in treating all septic bowel conditions, and has become convinced that if the remedy is given in proper doses it is a true intestinal antiseptic, but to obtain this effect we must avoid doses having an aperient action. He also noted similarly good effects in typhoid fever. The effect on the bowel was as in the other conditions, and as a secondary effect the temperature curve was lowered, and the whole disease assumed a more benign aspect.

"In some cases these good results did not follow, and the author suggests that perhaps in these cases there may have been some obscure alteration in the mucosa, interfering with the normal splitting up of the salt. Maberly is of the opinion that the antiseptic action is due to the setting free of oxygen during the process of chemical decomposition of the drug in the bowel. Experiments by Hay have already shown that sodium sulphate produces copious intestinal secretion, according to the amount of the dose and the strength of the solution. He showed further that the sulphate was split up in the intestines; the acid, being more easily absorbed than the base, disappeared partially from the small intestines, to return shortly afterwards. In the meantime the base was gradually undergoing absorption and excretion by the kidneys, and never pursued the same peculiar course of absorption and re-excretion as the acid. That portion of the salt which remained within the bowel unchanged caused the secretion of intestinal mucous fluid and consequent purgation, and not the portion absorbed into the blood."

---

According to the *Lancet* for November 17th, no less than eight English towns, viz., Brighton, Conway, Hackney, Lancaster, Macclesfield, Oldham, New Rommey, and Southwold, have elected medical men to the mayoralty.

---

L. Lapponi, M. D., physician to the present pope and his predecessor, died at Rome, December 17th, of cancer of the stomach with intercurrent pneumonia.

---

December, 1906, furnished the highest mortality rate of the decade in Chicago, with two exceptions, as 2,678 deaths were reported. The five chief causes of death were: Pneumonia, 450; consumption, 264; heart disease, 227; violence (including suicide), 213, and Bright's disease, 202.

---

Recent dispatches from Havana state that there are ten cases of yellow fever in the island, of which seven are in Havana, two in Cruces, and one in Guanabacoa.

---

At the annual meeting of the Floyd County Medical Society, held at Rome, on Friday evening, December 31st, the election of officers resulted as follows: President, Dr. J. W. Shaw; Vice-President, Dr. H. H. Battey; Secretary-Treasurer, Dr. W. L. Funkhauser; Delegate, Dr. J. W. Curry; Censors Dr. J. C. Watts, Dr. R. P. Cox, Dr. L. P. Hammond, of Rome.

---

Sir Victor Horsley, who has been connected with University College, London, as a student and as a teacher for about thirty years, has resigned his professorship of clinical surgery and his position as surgeon to the hospital on account of increasing professional duties.

